# Supplementary material for: High neural activity accelerates the decline of cognitive plasticity with age in Caenorhabditis elegans
Source: eLife. 2020 Nov 24;9:e59711. doi: 10.7554/eLife.59711 (PMC7685709; doi:10.7554/eLife.59711)
Supplement: Supplementary file 2. [file elife-59711-supp2.zip › Supplementary file 8. DrEdGe/how to access DrEdGE transcriptomics data.docx]

**Access transcriptomics data using DrEdGE**

DrEdGE must be served from a Web server to function. To run it on your local machine python must be installed.

- Open a terminal (command line interface)
- Change the directory to the one that contains the DrEdGE files
- Run one of the following commands in this directory:
- **Python 3:** python3 -m http.server 8006
- **Python 2:** python -m SimpleHTTPServer 8006

(to check which version of python is installed, type “python” in the command line)

- Then open the page <http://127.0.0.1:8006> in your browser
