# Supplementary material for: High neural activity accelerates the decline of cognitive plasticity with age in Caenorhabditis elegans
Source: eLife. 2020 Nov 24;9:e59711. doi: 10.7554/eLife.59711 (PMC7685709; doi:10.7554/eLife.59711)
Supplement: Supplementary file 2. [file elife-59711-supp2.zip › Supplementary file 8. DrEdGe/dredge-7.4.0-DESeq2/index.html]

DrEdGE
